# Distinct roles for interleukin-23 receptor signaling in regulatory T cells in sporadic and inflammation-associated carcinogenesis

**DOI:** 10.3389/fonc.2023.1276743

**Published:** 2024-02-05

**Authors:** Justin Jacobse, Jennifer M. Pilat, Jing Li, Rachel E. Brown, Aaron Kwag, Matthew A. Buendia, Yash A. Choksi, M. Kay Washington, Christopher S. Williams, Nicholas O. Markham, Sarah P. Short, Jeremy A. Goettel

**Affiliations:** ^1^ Department of Medicine, Division of Gastroenterology, Hepatology and Nutrition, Vanderbilt University Medical Center, Nashville, TN, United States; ^2^ Department of Pediatrics, Willem-Alexander Children’s Hospital, Leiden University Medical Center, Leiden, Netherlands; ^3^ Department of Pathology, Microbiology, and Immunology, Vanderbilt University Medical Center, Nashville, TN, United States; ^4^ Department of Medicine, Veterans Affairs Tennessee Valley Healthcare System, Nashville, TN, United States; ^5^ Program in Cancer Biology, Vanderbilt University School of Medicine, Nashville, TN, United States; ^6^ Medical Scientist Training Program, Vanderbilt University School of Medicine, Nashville, TN, United States; ^7^ Division of Pediatric Gastroenterology, Hepatology, and Nutrition, Monroe Carell Jr. Children’s Hospital at Vanderbilt, Vanderbilt University Medical Center, Nashville, TN, United States; ^8^ Center for Mucosal Inflammation and Cancer, Vanderbilt University Medical Center, Nashville, TN, United States; ^9^ Vanderbilt Institute for Infection, Immunology and Inflammation, Vanderbilt University Medical Center, Nashville, TN, United States

**Keywords:** colorectal carcinoma, interleukin-23, regulatory T cells, inflammation-associated cancer, sporadic cancer, macrophages, orthotopic MC-38, AOM-DSS

## Abstract

**Introduction:**

The pro-inflammatory cytokine interleukin-23 (IL-23) has been implicated in colorectal cancer (CRC). Yet, the cell-specific contributions of IL-23 receptor (IL-23R) signaling in CRC remain unknown. One of the cell types that highly expresses IL-23R are colonic regulatory T cells (Treg cells). The aim of this study was to define the contribution of Treg cell-specific IL-23R signaling in sporadic and inflammation-associated CRC.

**Methods:**

In mice, the role of IL-23R in Treg cells in colitis-associated cancer (CAC) was investigated using azoxymethane/dextran sodium sulphate in wild-type Treg cell reporter mice (WT, *Foxp3* ^YFP-iCre^), and mice harboring a Treg cell-specific deletion of IL-23 (*Il23r* ^ΔTreg^). The role of IL-23R signaling in Treg cells in sporadic CRC was examined utilizing orthotopic injection of the syngeneic colon cancer cell line MC-38 submucosally into the colon/rectum of mice. The function of macrophages was studied using clodronate. Finally, single-cell RNA-seq of a previously published dataset in human sporadic cancer was reanalyzed to corroborate these findings.

**Results:**

In CAC, *Il23r* ^ΔTreg^ mice had increased tumor size and increased dysplasia compared to WT mice that was associated with decreased tumor-infiltrating macrophages. In the sporadic cancer model, *Il23r* ^ΔTreg^ mice had increased survival and decreased tumor size compared to WT mice. Additionally, MC-38 tumors of *Il23r* ^ΔTreg^ mice exhibited a higher frequency of pro-inflammatory macrophages and IL-17 producing CD4^+^ T cells. The decreased tumor size in *Il23r* ^ΔTreg^ mice was macrophage-dependent. These data suggest that loss of IL-23R signaling in Treg cells permits IL-17 production by CD4^+^ T cells that in turn promotes pro-inflammatory macrophages to clear tumors. Finally, analysis of TCGA data and single-cell RNA-seq analysis of a previously published dataset in human sporadic cancer, revealed that *IL23R* was highly expressed in CRC compared to other cancers and specifically in tumor-associated Treg cells.

**Conclusion:**

Inflammation in colorectal carcinogenesis differs with respect to the contribution of IL-23R signaling in regulatory T cells.

## Introduction

Colorectal cancer (CRC) can be classified as sporadic or inflammation-associated carcinogenesis (IAC). A major risk factor for the development of IAC is inflammatory bowel disease (IBD). (1, 2) Duration of inflammation in both Crohn’s disease and ulcerative colitis over a patient’s lifetime determines the overall risk for developing colitis-associated cancer (CAC). (3) Of note, inflammation is not limited to CAC and is also a factor in sporadic CRC.

Strategies that prevent neoplastic transformation of polyps would yield the best outcome but requires a thorough mechanistic understanding of the cell types and pathways that drive tumorigenesis and foster precision-based therapeutic approaches to attenuate inflammation without compromising anti-tumor immunity. A major challenge is defining the specific pathways driving cancer progression under inflammatory conditions. Moreover, as the inflammatory milieu of sporadic CRC and CAC partially differs and is related to prognosis (4, 5), investigations to define the cancer-specific roles of select genes involved in the inflammatory process are needed.

In recent years, patients with IBD are being treated with monoclonal antibodies that block the pro-inflammatory cytokine interleukin (IL)-23 to induce remission. (6) However, the long-term consequence of blocking IL-23 signaling systemically and its impact on anti-tumor immunity is not known. *Il23r* is highly expressed on colonic regulatory T cells (Treg cells). (7) Moreover, targeting Treg cells or inhibiting their suppressive function is a promising treatment modality for CRC (8), but the role of IL-23R signaling in Treg cells in CRC has not been studied in a physiologically relevant cancer model (9).

Previously, we showed that IL-23R signaling in colonic Treg cells impairs their immunosuppressive function in part by increasing cell turnover. (10) With future therapies opting for targeted over systemic approaches, it will be important to define how signaling pathways operate in specific cell types for a given type of cancer to improve outcomes. Here, we investigated the role of IL-23R signaling in Treg cells in both sporadic and inflammation-associated CRC.

## Results

### Increased IL-23R expression in Treg cells during chronic intestinal inflammation

**Figure 1:**
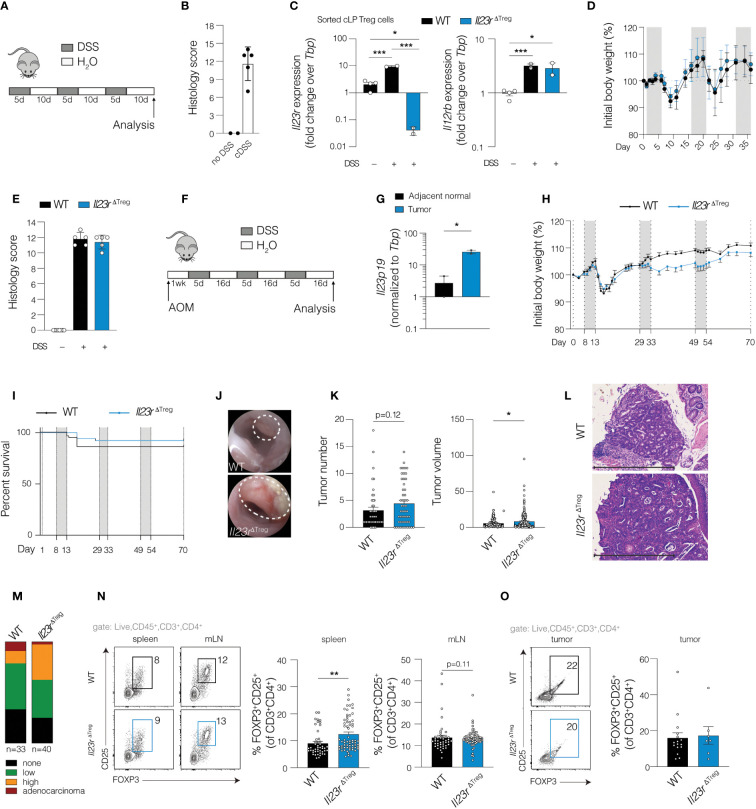
Cell-specific ablation of *Il23r* in Treg cells increases tumor volume and dysplasia in inflammation-associated carcinogenesis. **(A)** Schematic of chronic DSS model. **(B)** Composite colon histology score for mice on chronic DSS or water only control. **(C)** RT-qPCR of sorted colonic FOXP3^+^ cells from mice with IL-23R-sufficient Treg cells (*Il23r* ^flox/+^ *Foxp3* ^YFP-Cre^ *)* or IL-23R-deficient Treg cells (*Il23r* ^ΔTreg^) after chronic DSS or water only control. ANOVA with *post-hoc* Tukey. **(D)** Weight curves and **(E)** histology of WT (*Foxp3* ^YFP-Cre^) and *Il23r* ^ΔTreg^ mice during chronic DSS treatment. Data are representative of two independent experiments. **(F)** Schematic depicting mice AOM/DSS model of inflammation-associated carcinogenesis. **(G)** RT-qPCR for *Il23p19* expression in tumor and adjacent normal tissue in wild-type mice. Paired t-test. Data are representative from two independent experiments. **(H)** Weight curves of wild-type and *Il23r* ^ΔTreg^ mice subjected to AOM/DSS. Data are representative of five independent experiments. **(I)** Kaplan-Meier survival curves of AOM/DSS-treated mice. Data are pooled from five independent experiments, *N*=52-57. **(J)** Representative endoscopic images of tumors induced by AOM/DSS. **(K)** Quantification of tumor number per mouse and volume per tumor. Data are pooled from three independent experiments. **(L)** H&E-stained colon section depicting colon tumor with **(M)** grade of tumor dysplasia assessed by a pathologist. The grade shown is the highest grade of dysplasia per mouse. Chi-square among mice that developed dysplasia/tumors: *X^2^* (2, *N=56*) = 6.78, p=0.03. Data are pooled from three independent experiments. **(N)** Treg cell frequency in spleen, mesenteric lymph node (MLN) and **(O)** tumor with flow cytometry plots on the left and quantification on the right. Data are pooled from three independent experiments. **(N-O)** Unpaired T-test. *p<0.05; **p<0.01; ***p<0.001.

What induces Treg cells to express *Il23r* remains largely unknown. (11) Given that pro-inflammatory mediators such as IL-6 are involved in the polarization of naïve T cells towards IL-17 producing Th17 cells, which also upregulate *Il23r* for Th17 cell maintenance (12), we hypothesized that chronic inflammation may increase *Il23r* expression in colonic Treg cells. To experimentally test this, *Il23r* ^flox/+^ *Foxp3* ^YFP-iCre^ mice were subjected to three cycles of dextran sulfate sodium (DSS) for 5 days that were interrupted by a 10-day rest period each cycle to mimic the relapsing and remitting inflammation experienced by patients with IBD (**Figures 1A, B**). Reverse transcription-quantitative polymerase chain reaction (RT-qPCR) was performed on flow-sorted colonic Treg cells form DSS treated mice based on YFP expression. *Il23r* and *Il12rb1*, which forms a heterodimer with IL-23R and required for IL-23R signaling, were both increased following DSS treatment indicating that a functional IL-23R could be formed (**Figure 1C**). We then sought to determine if IL-23R signaling in Treg cells was required for chronic intestinal inflammation induced by DSS. To this end, we generated *Il23r* ^flox/flox^ *Foxp3* ^YFP-iCre^ mice (*Il23r* ^ΔTreg^) that lack IL-23R specifically in FOXP3^+^ Treg cells and compared these to IL-23R competent littermate *Foxp3* ^YFP-iCre^ mice (hereafter referred to as WT). At the experimental endpoint no changes in weight loss or colonic inflammation were observed by histology indicating that IL-23R signaling in colonic Treg cells is not required for chronic intestinal inflammation induced by chronic DSS (**Figures 1D, E**).

### IL-23R-deficient Treg cells promote tumor growth and dysplasia during inflammation-associated carcinogenesis

For patients with IBD, chronic inflammation contributes to an increased risk for developing intestinal carcinogenesis. Recent therapeutic strategies have targeted the IL-23R pathway to induce remission in patients with IBD. Although there is no evidence suggesting increased risk of CRC in these patients, IL-23 blockade is relatively new. Our recent work showed that colonic Treg cells unable to signal through IL-23R have increased capacity to restrict effector T cell responses and exhibit reduced cell turnover. (10) Thus, we hypothesized that within the tumor microenvironment, disrupting IL-23R signaling in Treg cells may interfere with anti-tumor immunity. To test this, we employed a model of inflammation-associated carcinogenesis (IAC) utilizing the mutagen azoxymethane (AOM) followed by repeated cycles of DSS (**Figure 1F**). We fist examined *Il23a* expression and found elevated expression in AOM/DSS tumors compared to adjacent normal tissue of WT mice (**Figure 1G**), consistent with a previous report (13) and likely the product of antigen presenting cells. (14) Following tumor induction with AOM/DSS in *Il23r* ^ΔTreg^ and WT mice, no differences in weight loss or survival were observed between groups (**Figures 1H, I**) while endoscopic examination confirmed tumor development in both groups (**Figure 1J**). At the experimental endpoint, tumor number was similar between groups while tumor volume of individual tumors was increased in *Il23r* ^ΔTreg^ mice (**Figure 1K**). Furthermore, a larger proportion of tumors exhibited high-grade dysplasia in *Il23r* ^ΔTreg^ compared to WT mice (**Figures 1L, M**). These findings suggest that the enhanced suppressive capacity and reduced turnover of IL-23R-deficient colonic Treg cells previously reported by our group (10) may restrict anti-tumor immunity in IAC.

To gain insight into the potential mechanism by which IL-23R-deficient Treg cells restrict anti-tumor immunity in IAC, we examined whether a numerical difference in Treg cells might drive the observed increase in tumor volume in *Il23r* ^ΔTreg^ mice. Our previous work established that the frequency of Treg cells in mLN and spleen was similar between *Il23r* ^ΔTreg^ and WT mice at baseline and only differed in the colon. (10) In contrast, following tumor induction with AOM/DSS, the frequency of Treg cells in the spleen and mLN was higher in *Il23r* ^ΔTreg^ mice, suggesting that intestinal inflammation can alter Treg cell frequency at other lymphoid compartments in an IL-23R-dependent manner (**Figure 1N**). Surprisingly, the frequency of Treg cells in AOM DSS-induced colonic tumors did not differ between *Il23r* ^ΔTreg^ and WT mice (**Figure 1O**). Finally, carcinogenesis in the AOM/DSS model is accompanied by the formation of tertiary lymphoid structures. (15) We assessed number of lymphoid aggregates and found no correlation with tumor number or genotype (**Supplementary Figure S1A**), nor did a survey of lymphoid aggregates indicate a difference in Treg cell frequency within the aggregates themselves (**Supplementary Figure S1B**). Collectively, these data suggest that a difference in Treg cell function or impact on another cell type rather than Treg cell frequency contributes to the difference in tumor volume between *Il23r* ^ΔTreg^ and WT mice.

**Figure 2:**
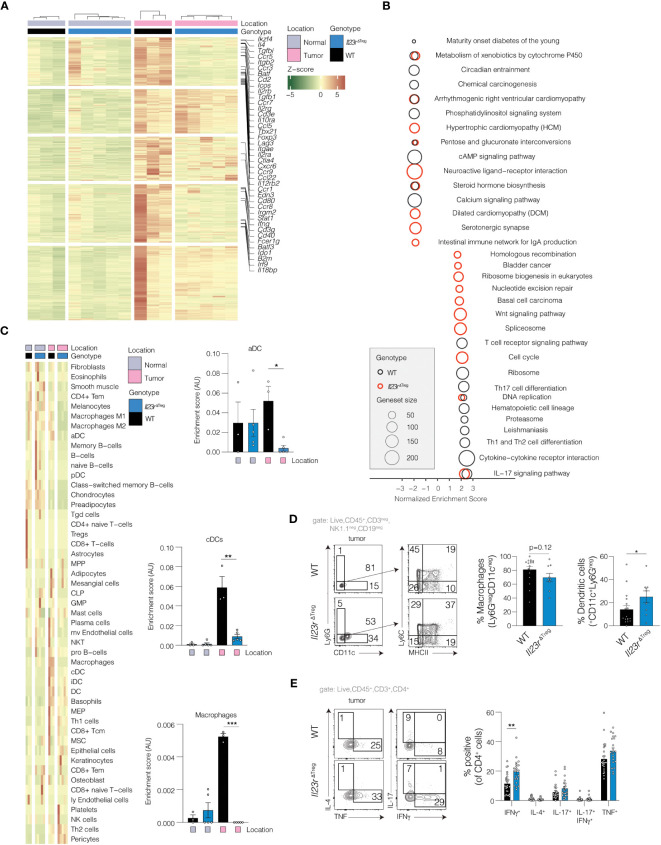
*Il23r* ^ΔTreg^ inflammation-associated colorectal tumors exhibit a pro-inflammatory transcriptional profile. **(A)** RNA-seq performed on tumors and adjacent normal tissue from AOM/DSS treated mice with heatmap demonstrating significantly upregulated genes in tumors from WT mice that were not upregulated in tumors from *Il23r* ^ΔTreg^ mice. Genes of interest are annotated. Z-scores were calculated based on normalized counts. Red indicates high z-scores for upregulated genes while green indicates high z-scores for downregulated genes. **(B)** Gene set enrichment analysis (GSEA) using Webgestalt. Two comparisons are shown: (1) tumor *vs*. adjacent normal in WT mice (black circles); (2) tumor *vs.* adjacent normal in *Il23r* ^ΔTreg^ mice (red circles). **(C)** PERMANOVA was used to identify cell types which were significantly different between tumors and adjacent normal tissue in WT and *Il23r* ^ΔTreg^ mice. Shown are the cell type enrichment scores calculated with xCell, with the significance indicated by the result of the SIMPER test for the comparison *Il23r* ^ΔTreg^ tumor *vs.* WT tumor. Only cell types that differed in at least one comparison are shown. **(D)** Flow cytometry of tumoral myeloid cells or **(E)** tumoral CD4^+^ T cells stimulated with PMA/ionomycin and stained for intracellular cytokines. **(D)** Mann-Whitney U-test. Lineage positive cells (CD3^+^, CD19^+^, NK1.1^+^) cells were excluded. **(E)** ANOVA with Dunnett’s *post hoc*. Data are pooled from two independent experiments. *p<0.05; **p<0.01; ***p<0.001.

To explore the potential impact of IL-23R-deficient Treg cells on other immune cell subsets within the tumor microenvironment, we defined transcriptional alterations within the tumor microenvironment of WT and *Il23r* ^ΔTreg^ mice by performing RNA-sequencing (RNA-seq) of tumor and tumor-adjacent colon tissue (hereafter adjacent normal). Differentially regulated genes were used to identify common and unique signatures of WT and *Il23r* ^ΔTreg^ tumors. Numerous cytokines and chemokines were upregulated in tumors of WT mice compared to adjacent normal tissue that were not altered when comparing *Il23r* ^ΔTreg^ tumors versus adjacent normal tissue (**Figure 2A**). Gene set enrichment analysis (GSEA) was employed to identify pathways differentially regulated in tumors of WT vs. *Il23r* ^ΔTreg^ mice (**Figure 2B**). Interestingly, pathways related to Th1, Th2, and Th17 cell differentiation as well as cytokine-receptor interactions were enriched in tumors of WT mice compared to *Il23r* ^ΔTreg^ mice. Conversely, a pathway uniquely upregulated in the tumors of *Il23r* ^ΔTreg^ mice was both canonical and non-canonical Wnt pathway genes. Considering the important role of Wnt signaling in epithelial stem cell biology and colorectal carcinogenesis, we quantified the number of OLMF4^+^ cells per high power field (hpf) as a proxy to enumerate intestinal stem cells. OLMF4 is a stem cell marker that has been reported to inhibit AOM/DSS-induced inflammation and crypt proliferation. (16, 17) No differences were observed in the number of OLMF4^+^ cells between WT and *Il23r* ^ΔTreg^ tumors (**Supplementary Figure S2A**). Collectively, these data suggest that differences in immune cell composition may contribute to the differences in tumor burden between WT and *Il23r* ^ΔTreg^ mice.

To delineate the cellular composition of AOM/DSS tumors, cell type analysis was performed computationally to extrapolate cell type identity from the RNA-seq data. After exclusion of cell types predicted to be absent from the tissue (**Supplementary Figure S2B**), 49 cell types remained (**Figure 2C**). Analysis using PERMANOVA showed that genotype contributed to the differences in cell composition between tumors. *Post-hoc* analysis indicated that dendritic cells and macrophages were decreased in tumors recovered from *Il23r* ^ΔTreg^ mice compared to tumors of WT mice. To corroborate the findings from the deconvolution analysis, flow cytometry of intratumoral dendritic cells (DCs) and macrophages was performed in independent validation experiments. This analysis showed that the frequency of intratumoral DCs (CD11c^+^CD45^+^CD3^neg^CD19^neg^NK1.1^neg^) was increased in tumors from *Il23r* ^ΔTreg^ mice, while the frequency of intratumoral macrophages (CD11c^neg^Ly6G^neg^CD45^+^CD3^neg^CD19^neg^NK1.1^neg^) trended lower in the tumors *Il23r* ^ΔTreg^ when compared to WT (**Figure 2D**). Thus, these results were partially concordant with the deconvolution analysis and suggest that the absence of IL-23R signaling in Treg cells decreases intratumoral macrophages during AOM/DSS-mediated carcinogenesis. Finally, we examined the regulation of the intratumoral effector CD4^+^ T cell response in absence of IL-23R-signaling in Treg cells and found that effector T cells produce more IFNγ in absence of IL-23R signaling in Treg cells (**Figure 2E**). Altogether, these data show that IL-23R signaling in Treg cells restricts AOM/DSS-mediated carcinogenesis, potentially via regulation of intratumoral macrophages and/or cytokine production by CD4^+^ effector T cells.

### IL-23R-deficient Treg cells inhibit tumorigenesis in a sporadic model of carcinogenesis in a macrophage-dependent manner

**Figure 3:**
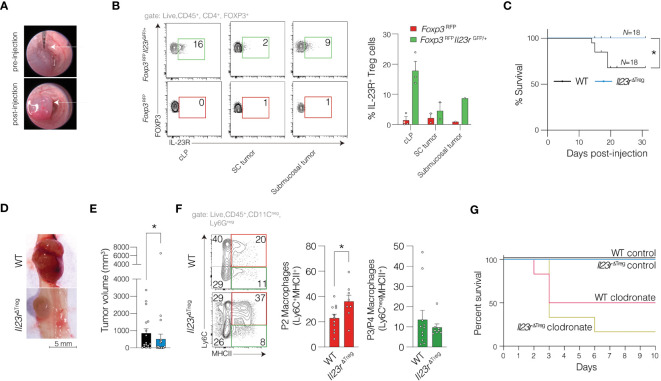
IL-23R-signaling in Treg cells protects against sporadic colorectal cancer in an orthotopic model. **(A)** MC-38 cells were submucosally injected into the colon of mice with representative photo of colon lumen pre- and post-injection. **(B)** MC-38 cells were submucosally or subcutaneously injected into *Foxp3* ^RFP^ and *Foxp3* ^RFP^ *Il23r* ^GFP/+^ mice with data pooled from three independent experiments to assess for expression of IL-23R in Treg cells. **(C)** Kaplan-Meier survival curve for WT and *Il23r* ^ΔTreg^ mice submucosally injected with MC-38 cells (as in **A**). Mantel-Haenszel statistic. **(D)** Representative macroscopic image and **(E)** quantification of tumor volume per mouse across multiple independent experiments. Mann-Whitney U-test. **(F)** Representative flow cytometry plot of intratumoral macrophages (left) with quantification (right). Data are pooled from six independent experiments. Unpaired t-test. *p<0.05. **(G)** Kaplan-Meier survival curve for WT and *Il23r* ^ΔTreg^ mice submucosally injected with MC-38 cells with some mice being depleted of macrophages using clodronate while a separate cohort treated with control liposomes. Data are pooled from two independent experiments, *N=4* (clodronate treated mice) and *N=6* (control liposome treated mice).

Since the majority of CRC cases are sporadic, we also sought to examine the role of IL-23R signaling in Treg cells in sporadic CRC. Prior work has utilized MC-38 cells injected subcutaneously (SC).(9) However, to employ a more relevant *in vivo* setting, we performed endoscope-guided orthotopic injections of MC-38 cells into the colonic submucosa (**Figure 3A**). We then generated *Foxp3* ^RFP^ *Il23r* ^GFP/+^ mice (IL-23R Treg reporter) and validated IL-23R protein expression using *in vitro* induced Th17 cells (not shown). We then determined expression of IL-23R protein in Treg cells in colon tissue and MC-38 tumors injected SC or orthotopically (**Figure 3B**). We then compared tumor development and burden of submucosally injected MC-38 tumors cells in WT and *Il23r* ^ΔTreg^ mice two weeks following injection of MC-38 cells. The survival of WT mice was lower compared to the survival of *Il23r* ^ΔTreg^ mice (**Figure 3C**) with tumor volume in *Il23r* ^ΔTreg^ mice lower compared to tumor volume in WT mice (**Figures 3D, E**). Interestingly, this was in contrast to our findings in the inflammation-associated carcinogenesis model. Since transcriptional analysis and flow cytometry implicated antigen presenting cells as being differentially regulated in *Il23r* ^ΔTreg^ mice following AOM/DSS, we assessed pro-inflammatory macrophages (Ly6C^+^MHCII^+^) by flow cytometry and found the frequency of intratumoral pro-inflammatory macrophages to be increased in *Il23r* ^ΔTreg^ mice (**Figure 3F**). To determine whether MC-38 tumors are larger in *Il23r* ^ΔTreg^ mice due to macrophages, we depleted macrophages via intraperitoneal clodronate injection and orthotopically injected these mice with MC-38 tumors cells. However, due to clodronate-attributed mortality no conclusion could be drawn regarding the macrophage dependency of the orthotopic MC-38 model (**Figure 3G**). Given that *Il23r* ^ΔTreg^ mice have an increased frequency of colonic Treg cells that are also more suppressive than colonic Treg cells from WT mice (10), we anticipated that intratumoral Treg cells may be increased in the tumor MC-38 model. However, the frequency of intratumoral Treg cells did not differ between *Il23r* ^ΔTreg^ and WT mice (**Supplementary Figure S3A**). Collectively, these data suggest that IL-23R-deficient Treg cells protect mice from MC-38-mediated sporadic colorectal carcinogenesis, likely via increased intratumoral pro-inflammatory macrophages that promote anti-tumor immunity.

Previous work shows that IL-23R signaling in Treg cells in SC-injected MC-38 cells inhibit IFNγ production by CD8^+^ T cells. (9) Using a similar approach, we quantified cytokine production following orthotopic injection of MC-38 cells and observed a decreased IFNγ production by CD4^+^ cells that approached statistical significance, but no increase was observed in CD8^+^ T cells (**Supplementary Figures S3B–C**). Similarly, an increase in the frequency of IL-17^+^ CD4^+^ T cells also approached statistical significance in *Il23r* ^ΔTreg^ mice compared to WT mice (**Supplementary Figure S3C**). The frequency of intratumoral dendritic cells and neutrophils was not altered between WT and *Il23r* ^ΔTreg^ mice (**Supplementary Figure S3D**).

Since the injection medium utilized with the orthotopic model differs from traditional SC injections, we mirrored the orthotopic model by injecting MC-38 cells SC into *Il23r* ^ΔTreg^ and WT mice. Here we observed a decrease in tumor volume from *Il23r* ^ΔTreg^ mice compared to WT mice similar to the orthotopic model (**Supplementary Figure S4A**). However, in contrast to the lower CD4^+^ T cell IFNγ production observed in *Il23r* ^ΔTreg^ mice orthotopically injected with MC-38 cells, in SC MC-38 cell tumors, there was no significant difference in IFNγ production by CD4^+^ or CD8^+^ T cells between genotypes (**Supplementary Figures S4B–C**). Similarly, no differences in the frequency of intratumoral Treg cells were observed (**Supplementary Figure S4D**). To further define the immune cell tumor microenvironment, we quantified the frequency of myeloid cells including dendritic cells, neutrophils, and macrophages. While the frequency of intratumoral dendritic cells and neutrophils between WT and *Il23r* ^ΔTreg^ mice was similar (**Supplementary Figure S4E–F**), the frequency of intratumoral pro-inflammatory macrophages was increased in *Il23r* ^ΔTreg^ mice compared to WT mice (**Supplementary Figure S4G**). Finally, to determine the role of macrophages in driving enhanced anti-tumor immunity in *Il23r* ^ΔTreg^ mice, we depleted macrophages in the SC model using paratumoral injections as previously described. (18) Interestingly, depletion of macrophages increased MC-38 tumor size in *Il23r* ^ΔTreg^ mice whereas the tumor size in WT mice treated with clodronate was not statistically significant different from WT mice treated with control liposomes (**Supplementary Figure S4A**). Thus, IL-23R-deficient Treg cells inhibit tumorigenesis in the subcutaneous sporadic MC-38 model of carcinogenesis in a macrophage-dependent manner.

### Expression of IL-23R is increased in tumor-associated Treg cells in human colon cancer

**Figure 4:**
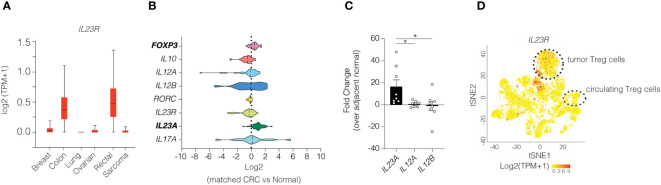
Colonic Treg cells in human colon tumors express high levels of *IL23R.* **(A)** *IL23R* expression among various cancer types. **(B)** Transcriptional analysis of select genes in matched samples from patients with colorectal cancer obtained from TCGA. **(C)** RT-qPCR of the *IL23* subunits in patients with colorectal cancer. ANOVA with *post-hoc* Tukey. * *p*<0.05 **(D)** Re-analysis of single cell RNA-seq dataset for *IL23R* expression in various immune cells. (19) Heatmap indicates z-scores for expression with red indicating higher expression. Cluster designations were implemented as described in the published manuscript.

Our prior investigations in mice show that IL-23R signaling in Treg cells impairs their suppressive function of effector T cells and leads to increased Treg cell turnover. (10) Given the poor prognosis associated with increased tumor Treg cells in many solid tumors in humans, we sought to investigate whether IL-23R signaling in Treg cells may be active in this setting. To explore this possibility, we surveyed data obtained from The Cancer Genome Atlas (TCGA) to examine *IL23R* expression across various cancer types and found that *IL23R* expression was increased specifically in colon and rectal cancers when compared to other cancer types (**Figure 4A**). We subsequently focused on sporadic CRC as this form of CRC is more prevalent than IAC in humans. By comparing paired tumor and adjacent normal tissue samples from 24 individuals with colon cancer, we found transcript levels for the IL-23-specific subunit *IL23A* and *FOXP3* were both elevated in tumor tissue compared to adjacent normal tissue (**Figure 4B**). The increase in tumor *IL23A* was corroborated in a validation cohort via qPCR using patient-matched tumor and non-tumor biopsies from 9 CRC patients at Vanderbilt University Medical Center (**Figure 4C**). Since whole tissue expression of *IL23R* does not provide the cell type-specific resolution, we analyzed a CRC single-cell RNA-seq dataset (19) that included cells from 12 patients. In this cohort we found *IL23R* expression to be readily detectable in tumor Treg cells but not in circulating Treg cells (**Figure 4D**). Taken together, this suggest that both Treg cells and IL-23 are increased within colon tumors and highlights the importance of evaluating tumor resident cells.

Given the increase in *IL23A* transcripts in tumors, it is reasonable to consider the possibility that IL-23 may act directly on tumor epithelial cells to promote tumor development as intestinal epithelial cells also express *IL23R*. To explore this possibility, we treated generated human organoids derived from healthy colon or CRC tumors and treated with exogenous IL-23 and measured the impact on cell growth. In this *ex vivo* model, IL-23 did not potentiate organoid growth after 3 days in culture (**Supplementary Figure S5A**). Since this may have been an artifact of the *ex vivo* culture system, we examined activation the downstream target of IL-23R (i.e., STAT3) in patient tumors. Using a tumor array containing intestinal tissue biopsies from CRC patients and adjacent normal tissue, we performed immunohistochemical staining for phospho-STAT3 (pTyr705). Activated STAT3 was readily detected in non-epithelial cells of tumor sections, but not adjacent normal tissue (**Supplementary Figure S5B**). While these data would be consistent with IL-23R pathway activation in tumor tissue, STAT3 activation can also be mediated by IL-6 or IL-10. It is noteworthy, however, that expression of *IL6* and *IL10* was similar between tumor and adjacent normal tissue (data not shown). Collectively, these data support the idea that IL-23R signalling in Treg cells may antagonize the normal suppressive function of Treg cells to promote anti-tumor immunity in CRC.

## Discussion

The pro-inflammatory cytokine IL-23 is present in early CRC and throughout CAC; however, IL-23 signaling has been described as both pro- and anti-tumorigenic. (20, 21) Although Treg cells have long been associated with poor prognosis in solid tumors, the role of IL-23R signaling in Treg cells during CRC has not been well characterized. Here, we found that IL-23R signaling in Treg cells plays opposing roles in murine models of sporadic versus inflammation-associated CRC. In inflammation-associated CRC, IL-23R signaling in Treg cells inhibits carcinogenesis, whereas in sporadic CRC, IL-23R signaling in Treg cells promotes carcinogenesis. These data highlight that the circumstances and conditions that give rise to colon cancer likely have distinct functional contributions and choice of targeted therapeutics will need to consider these differences.

Previous studies have shown macrophage-derived IL-23 to be present in SC MC-38 tumors. (22) In addition, IL-23R is upregulated in Treg cells isolated from SC MC-38 tumors (9, 22) Signaling via IL-23R increases STAT3 phosphorylation in Treg cells making them more sensitive to IL-12-induced STAT4 phosphorylation. (9, 22) Whether these observations altered susceptibility to an orthotopic MC-38 cell model was unknown. Orthotopic models represents a more physiologically and immunologically relevant environment that may be more readily translational to humans. Our study shows that, IL-23R signaling in Treg cells significantly promotes colonic MC-38 tumorigenesis. However, we found that not all observations from the SC tumor models using MC-38 cells could be directly translated to an orthotopic system. The increased frequency of IFNγ^+^ CD8^+^ T cells, that we and others observed in *Il23r* ^ΔTreg^ mice, only occurred in SC-injected tumors. (9) In contrast, we did not observe differences in IFNγ^+^CD8^+^ cell frequency in the orthotopic model. With location of injection being the only variable between the two, the local environment likely has a greater impact on the functional role tumor infiltrating immune cells play and highlights the importance of utilizing a physiologically relevant tumor microenvironment when possible.

Understanding how tumor-infiltrating Treg cells alter the tumor microenvironment might yield new or improved therapeutic options for CRC as others have demonstrated (23, 24). For instance, how does IL-23R signaling in Treg cells promote orthotopic MC-38 tumorigenesis? One explanation may lie with tumor macrophages and the regulation of cytokine production by CD4^+^ T cells. Tumor-associated macrophages (TAMs) have been observed to be anti-inflammatory or pro-inflammatory depending on cancer type and stage (as reviewed elsewhere (25, 26). In our study, intratumoral macrophage frequency inversely correlated with tumor size in the AOM/DSS and the MC-38 model and was altered in *Il23r* ^ΔTreg^ mice. Treg cells are immunosuppressive and Treg cell subsets, particularly RORγt^+^ Treg cells, have been demonstrated to suppress correlating subsets of conventional T cells such as IL-17 producing Th17 cells. (7) Since IL-23R signaling stabilizes RORγt expression, the lack of *Il23r* in Treg cells of *Il23r* ^ΔTreg^ mice likely contributes to greater Th17 cell differentiation during inflammation. (10) Pro-inflammatory macrophages have been shown to respond to IL-17 by producing cytokines, including IL-12 (27), that in turn stimulate T cell effector functions. Future experiments are needed to determine whether the absence of IL-23R signaling in Treg cells and expanded IL-17-producing CD4^+^ T cells promotes pro-inflammatory macrophage function to reduce tumorigenesis.

Interestingly, IL-23R deletion in Treg cells in the AOM/DSS model of CAC increased tumor size and dysplasia but did not affect tumor number. This raises the possibility that IL-23R signaling in Treg cells affects tumor progression rather than initiation in this model. Importantly, the increased tumor size in *Il23r* ^ΔTreg^ mice was not due to DSS alone as DSS-treated WT and DSS-treated *Il23r* ^ΔTreg^ mice developed i.e. a similar degree of inflammation based on histology. The increased tumorigenesis observed in *Il23r* ^ΔTreg^ mice differs from an earlier report, in which mice transplanted with bone marrow from IL-23-deficient mice (*Il23p19^-/-^*) exhibited decreased tumor growth. (14) However, other cell types are competent to respond to IL-23, including group 3 innate lymphoid cells and intraepithelial lymphocytes. In colon cancer ILC3s are impaired due to increased transitioning to ILC1-like cells that is mediated in part by IL-23. (28) Thus global IL-23 deficiency would be expected to limit this ILC1 transitioning and supports the need to evaluate pathways in a cell-specific manner to understand how these cells function in tumorigenesis.

Increased IL-23R expression in Treg cells appears to be similar in both murine models and human sporadic CRC. Notably, our analysis of TCGA data suggests that elevated *IL23R* expression is specific to CRC compared to other cancer types. Our results indicate that IL-23R signaling in colon tumors is active in non-epithelial cells, likely infiltrating immune cells, but the causal effects of Treg cell-specific IL-23R signaling requires additional studies in humans to understand the implications in CRC pathogenesis. One limitation of this work is that we did not study the suppressive function of IL-23R signaling in tumor infiltrating Treg cells in human CRC. Moreover, we have not performed functional characterization of tumor-associated Treg cells. We have recently generated *Fox3* ^RFP^ *Il23r* ^GFP^ mice to permit this analysis in future studies. Another limitation is that we utilized the well-characterized MC-38 CRC cell line, which does not recapitulate spontaneous carcinogenesis and is restricted to mice on the C57BL/6 background. A spontaneous colorectal carcinogenesis model (*CDX2^Cre^*; *Apc^Δ^* ^580/+^) has reported IL-23 expression in early adenomas, and global deletion of IL-23 reduces sporadic tumors in these mice. (29) It would be interesting to evaluate the role for IL-23-dependent signals using a spontaneous model where the onset of colorectal tumors can be timed such as the *Lrig1* ^CreERT2/+^; *Apc* ^flox/+^ system (30).

In conclusion, we show that the immunological tumor micro-environment is important for evaluating contributions of specific genes in tumorigenesis and should be considered when selecting *in vivo* models. The role of IL-23R signaling in Treg cells in CRC appears dichotomous: pro-tumorigenic in sporadic CRC and anti-tumorigenic in CAC. Consistent with IL-23 impairing Treg cell function in the intestine, blocking IL-23R signaling specifically in Treg cells in a CAC model increased tumor volume and dysplasia, perhaps by antagonizing anti-tumor immunity. In a sporadic CRC model, targeting IL-23R in Treg cells increased IL-17 production by CD4^+^ T cells and increased the frequency of pro-inflammatory macrophages that was associated with decreased tumor volume and improved survival. Selective targeting of IL-23R may be a promising therapeutic approach for sporadic CRC but may have undesirable effects in CAC.

## Materials and methods

### Mice

C57BL/6 *Foxp3* ^YFP-iCre^ (The Jackson Laboratory, Bar Harbor, ME, stock #016959, designated WT in the text), *ll23r* ^ΔTreg^, lacking IL-23R in FOXP3^+^ Treg cells, *Foxp3* ^RFP^ (Jackson, stock #008374) and *Il23r* ^GFP^ (Jackson stock #035863) mice were used. *Il23r* ^ΔTreg^ mice have been previously described. (10) Mice were housed in a specific-pathogen free vivarium at Vanderbilt University Medical Center (VUMC). All experiments were performed using 6–15-week-old female or male mice unless otherwise indicated. Littermates were used where possible. Experiments were approved by the VUMC Institutional Animal Care and Use Committee.

### Inflammation-associated carcinogenesis

7-8-week-old mice were co-housed, bedding was mixed 14 days prior to the start of the protocol, and 3 days prior to start of the protocol mice were placed on deprivation caps to acclimate mice to drinking exclusively from water bottles. Mice were intraperitoneally injected with 10 mgkg^-1^ azoxymethane (Sigma-Aldrich) and exposed to three 5-day cycles of 3.5% dextran sodium sulphate (TdB consultancy, Uppsala, Sweden). Each DSS cycle was followed by a 16-day recovery period of regular autoclaved water.

### MC-38 model of sporadic cancer

MC-38 cells were grown in DMEM with high glucose and pyruvate (Gibco, NY, #11995065) supplemented with 10% FBS and 100 U/mL pen/strep. MC-38 cells were confirmed to be pathogen-free by the Vanderbilt Translational Pathology Shared Resource prior to *in vivo* use. All experiments were performed with cells from the same passage (passage 34). For injections, cells were suspended in 40% PBS, 50% DMEM, and 10% matrigel (Corning, NY, #356231). 5x10^4^ cells in 50 uL were injected SC into the right flank or colonoscope-guided orthotopically injected into the mucosal lining of the distal colon (31).

### Tumor measurements

At euthanasia, images were taken using a SMZ1270 dissecting scope (Nikon, Melville, NY) in concert with NIS Elements version 5 (Nikon). Tumors were located and identified by vascular, non-dimpled appearance from these images in conjunction with direct visualization. Tumors were measured macroscopically in two perpendicular dimensions using digital calipers, and tumor volume was calculated using the previously validated formula W2*L/2, where W (width) was the shorter caliper measurement, and L (length) was the longer caliper measurement (32).

### Macrophage depletion

Macrophage depletion was done with clodronate (brand name clodrosome) and compared to mice injected with control liposome (both from Encapsula NanoSciences, TN). For the orthotopic MC-38 model, mice were maintained on enrofloxacin water (0.8 mgmL^-1^) and injected with 100 uL clodronate or control liposomes one day prior or one the same day as MC-38 injection followed by a second injection three days after the first injection. For the subcutaneous MC-38 model, mice were injected with 50 uL clodronate or control liposomes every other day with the first injection on the same day as the MC-38 injection.

### Histology

Hematoxylin and eosin (H&E) staining was performed by the Vanderbilt Translational Pathology Shared Resource on formalin-fixed paraffin-embedded (FFPE) colon sections or a custom tumor micro-array. For IHC, slides were placed on the Leica Bond IHC stainer. All steps besides dehydration, clearing, and coverslipping were performed on the Bond stainer. Slides were deparaffinized and hydrated. IHC for OLFM4 was done by FR with the following protocol: (1) Antigen Retrieval: Citrate buffer pH 6.0, decloaking chamber 105°C for 15 minutes, 10 minute bench cool down; (2) Peroxidase Block: 0.03% H_2_0_2_ with sodium azide, 5 minute incubation; (3) Primary Antibody (Cell Signaling, 39141, Massachusetts): 1:500 dilution, overnight incubation; (4) Detection: Dako (K4003) Envision + HRP Labelled Polymer, 30 minute incubation; (5) Chromogen: DAB^+^, 5 minute incubation. All incubations are at room temperature. Scoring of histology was performed by pathologist (MKW) who was blinded to experimental conditions.

### Tumor digestion

Tumors were isolated, minced, and placed into 25 mL prewarmed RPMI 1640 media containing 0.1 mgml^-1^ Liberase TL (Roche, Basel, Switzerland), 0.05% DNAse I (Sigma-Aldrich, D5025) and 20 mM HEPES and shaken at 37°C for 60 minutes in a non-CO_2_ MaxQ4450 horizontal shaker (Thermo Fisher Scientific, Waltham MA). Cells were pulled through a 10 mL syringe 20 times and filtered through a 70-μm cell strainer into an equal volume of cold RPMI 1640 media containing 5% FBS, 0.05% DNAse I, 20 mM HEPES on ice. Cells were spun for 10 minutes at 4°C and 475 g and resuspended in 40% Percoll (Sigma-Aldrich) solution and underlaid using 90% Percoll. The 40/90 gradient was spun for 25 minutes at 20°C at 475 g with no brake or acceleration. The interphase layer was recovered and washed in fluorescence cytometry (FC) buffer (PBS w/o Ca2^+^/Mg2^+^ containing 2% FBS and 2 mm EDTA) and spun again for 10 minutes at 20°C and 475 g prior to downstream application(s).

### Whole slide imaging

Images from whole slides were captured using a high-throughput Leica SCN400 Slide Scanner automated digital image system from Leica Microsystems. Whole slides were imaged at 40X magnification to a resolution of 0.25 µm/pixel. Images were also taken with a EVOS XL Core followed by adjustment of brightness and contrast using Adobe Photoshop (equal for all images).

### RNA isolation

Extraction of RNA from whole tissue or organoids was performed on tissue stored in RNA-later (Sigma-Aldrich) until homogenization using a Tissue-Tearor (Dremel, Racine, WI), followed by phenol/chloroform extraction as described (33) and clean-up with the Rneasy Mini Kit with on-column DNAse digestion according to manufacturers’ instructions.

### RNA-sequencing

Quality control for RNA was performed by the Vanderbilt Technologies for Advanced Genomics core using RNA 6000 Pico (Agilent, Santa Clara CA), and cDNA library preparation using a NEB library preparation kit. Paired end 150bp sequencing was performed on a NovaSeq 6000 (Illumina, San Diego, CA).

### RNA-sequencing

Samples were trimmed with fastp (version 0.20.0) using default parameters. Quantification was performed using Salmon (version 1.4.0) against a decoy transcriptome (Mus musculus Gencode version 21). Further analysis was performed in R (version 4.1.2) in R studio (version 2021.09.2 + 382). For differential expression analysis, limma (version 3.50.3) was used on log-CPM transformed counts with prior count set to 3 or DESEq2 (version 1.34.0) was used on non-normalized counts. Unless otherwise indicated, genes with an adjusted p value <0.05 (-log_10_ of 0.05 is 1.3) and log_2_ fold change >|1| were considered differentially regulated. For heatmaps with gene counts, a Z-score, i.e. the number of standard deviations above or below the mean, of normalized counts was calculated using scale function in R.

For GSEA using Webgestalt, the web-based version accessible at webgestalt.org was utilized with their default parameters. The input was a list of all genes sorted on fold change in increasing order generated using Limma. Resulting normalized enrichment scores (NES), which reflect the degree to which a gene set is overrepresented at the extremes of the entire ranked list (34) and size of the gene sets were plotted. Annotation was done with AnnotationDbi (version 1.56.2) using org.Mm.eg.db (version 3.14.0). Images were generated with pheatmap (version 1.0.12), complexheatmap, RColorBrewer (version 1.1-3), ggplot2 (version 3.3.5), and EnhancedVolcano (version 1.12.0).

### Cell type analysis

For cell type analysis (also known as deconvolution) xCell was used following the instructions from the authors of the package. (35) Cell types with a p-value cutoff of 0.20 in a beta distribution were excluded from the analysis, as this suggested that these cell types were not present in the tissue. Then, cell type scores were recalculated. An arbitrary cutoff of 4 samples was used to determine whether gene expression related to a certain cell type was present, i.e. if the gene expression associated with a cell type was present in less than 4 samples (in any group), the cell type was excluded from analysis to increase the specificity of the cell types that were present.

Since the data were not normally distributed as determined by the multivariate normality test (using mvnormtest), a permutational analysis of variance (PERMANOVA, Bray-Curtis, 999 permutations) was used to examine contribution of genotype and tissue (tumor or normal). To this end, genotype and tissue were combined into a composite variable with 4 groups. *Post-hoc* tests were done with a multilevel pairwise comparison (pairwise adonis, version 0.4.1) and corrected for multiple comparisons according to Benjamin-Hochberg. Adjusted p<0.05 was considered statistically significant. Finally, for the multiple comparisons that differed, Similarity Percentages (SIMPER) analysis was used to examine cell types contributing to dissimilarity. Again, adjusted p<0.05 was considered statistically significant.

### Flow cytometry

To stimulate T cells, single cell suspensions were incubated for 5 hours at 37°C (5% CO_2_) with 50 ngmL^-1^ phorbol myrisate acetate (PMA) and 1 μgmL^-1^ ionomycin (Biolegend, California) in the presence of Golgi-Stop (BD) for the last 4 hours. For cell surface staining, samples were blocked using 30 mL normal rat serum (StemCell Technologies) and incubated in the antibody cocktail for 20 minutes at 4°C in the dark. Intracellular cytokine staining was performed using Cytofix/Cytoperm (BD, New Jersey) according to manufacturer’s instructions. Flow cytometric analysis was performed using a 4-Laser Fortessa, 5-laser LSRII (BD) with FACSDiva software (BD), or a Cytek Aurora (Cytek, CA). Fluorescence-activated cell sorting (FACS) was performed on a FACS Aria III (BD). Analyses were performed using FlowJo (BD Biosciences). For all flow experiments, a live/dead stain (ThermoFisher) was used to only assess live cells. Antibodies used for flow cytometry or cell sorting are listed in **Supplementary Table S1.**

### Human organoid cultures

Deidentified human colorectal tumor and adjacent normal colon tissues were collected at Vanderbilt University Medical Center and provided by the NCI Cooperative Human Tissue Network in accordance with the Vanderbilt Institutional Review Board. Organoids were established in accordance with published methods. (36) Briefly, for tumor organoids, minced tumor tissue was incubated in digestion buffer (Advanced DMEM F12, Gibco; 10% FBS; 100 U/mL penicillin; 100 μgmL^-1^ streptomycin; 0.125 mgmL^-1^ Dispase II, Roche; 0.1 mgmL^-1^ collagenase XI; Sigma-Aldrich) for 1 hour at 37°C. Following digestion, the supernatant containing digested tumor fragments was collected, washed, and suspended in growth factor reduced Matrigel (356231, Corning). For normal colon organoids, intestinal mucosa was minced, and tissue fragments were then incubated in chelation buffer (2 mM EDTA in phosphate-buffered saline) for 30 minutes at 4°C before 2 minutes of gentle shaking to free intestinal crypts. Crypts were collected and suspended in growth factor reduced Matrigel (Corning). Human organoids were overlaid in Advanced DMEM F12 media containing 50% LWRN-cell (ATCC) conditioned media, B27 (ThermoFisher), N2 (ThermoFisher), 100 U/mL pen/strep (Gibco), 1% HEPES (Corning), 1% Glutamax (Gibco), 1 mM N-acetylcysteine (A9165, Sigma), 50 ngml^-1^ EGF (2028EG200, R&D), 500 nM A-83-01 (2939, Tocris), 10 μM SB202190 (S7067, Sigma), 10 mM nicotinamide (Sigma), 10 nM [Leu15]-Gastrin I (G9145, Sigma), 100 μgmL^-1^ Primocin (Invivogen), and 3 μM CHIR99021 (4423, Tocris, for colon organoids only). To assess changes in size, human tumor and colonic organoids were split by ezymatic digestion (TrypLE, Gibco) or manual dissociation by vigorous pipetting, respectively, and replated. Fields of organoids were imaged, and the same fields were imaged again after 3 days. Size for all images was quantified in ImageJ by an investigator blinded to treatment status.

### RT-qPCR

cDNA was synthesized from 1 µg of RNA using qScript XLT cDNA SuperMix (Quantabio, Massachusetts). RT-qPCR was performed in technical duplicates with TaqMan™ probes (ThermoFisher, Massachusetts) and TaqMan™ Universal PCR Master Mix (ThermoFisher, Massachusetts). RT-qPCR results were analyzed by the delta-delta Ct method and normalized to *Gapdh*. For RT-qPCR of mouse sorted Treg cells the methods previously described were used (10).

### TCGA analysis

Data for **Figure 4A** was retrieved using the web interface from UALCAN (37). For **Figure 4B**, expression levels were queried from Illumina HiSeq and Illumina GA RNASeqV2 data in The Cancer Genome Atlas [TCGA(38)] colon adenocarcinoma (COAD) data set (*N*=264 colorectal cancer) and normalized to expression levels observed in adjacent normal colon (*N*=39 normal colon). Only patients that had both tumor and adjacent normal were included in the latter analysis. No statistics were done.

### Statistics

Details regarding the statistical analyses are indicated in the figure legends. *N* represents biological replicates, unless otherwise indicated. Data shown are representative or pooled data from at least two independent experiments with similar results. RNA-sequencing (RNA-seq) was performed on multiple samples from one experiment. The sequence of sample processing was counterbalanced. Age-, sex- and, where feasible, littermate-matched mice were used. Cage-effects were examined. Apart from bulk RNA-seq, statistical analyses were performed using GraphPad Prism (9.1.2). Other than RNA-seq, graphs were made in GraphPad Prism and edited with Adobe Illustrator. Sample size was determined empirically. Outliers were not removed except for **Figure S4A**, in which one outlier was removed from the WT control mice and one outlier was removed from the *ll23r* ^ΔTreg^ control mice. Positive cells in IHC images were detected using QuPath (0.4.3) using the “positive cell detection” function, except for OLMF4, which was counted manually by two blinded investigators.

## Data availability statement

The datasets presented in this study can be found in online repositories. The names of the repository/repositories and accession number(s) can be found below: https://www.ncbi.nlm.nih.gov/geo/, GSE240707. Scripts to analyze data have been published before (39).

## Ethics statement

The studies involving humans were approved by the Vanderbilt Institutional Review Board. The participants provided their written informed consent to participate in this study. The animal studies were approved by the VUMC Institutional Animal Care and Use Committee. All studies were conducted in accordance with the local legislation and institutional requirements.

## Author contributions

JJ: Data curation, Formal analysis, Investigation, Methodology, Project administration, Validation, Visualization, Writing – original draft, Writing – review & editing. JP: Investigation, Writing – review & editing. JL: Investigation, Writing – review & editing. RB: Investigation, Methodology, Writing – review & editing. AK: Investigation, Writing – review & editing. MB: Investigation, Writing – review & editing. YC: Investigation, Writing – review & editing. MW: Formal analysis, Investigation, Writing – review & editing. CW: Writing – review & editing. NM: Investigation, Methodology, Resources, Writing – review & editing. SS: Investigation, Methodology, Resources, Writing – review & editing. JG: Conceptualization, Funding acquisition, Methodology, Resources, Supervision, Visualization, Writing – original draft, Writing – review & editing.

## Supplementary material

Supplementary Figure 1 Frequency of Treg cells in spleen and mLN is increased in *Il23r* ^ΔTreg^ mice following AOM/DSS. **(A)** Mice were injected with AOM/DSS as described in with quantification of colonic lymphoid aggregates (LA) shown. **(B)** Representative flow cytometry plots of Treg cells in LA (left) and frequencies (right). Unpaired t-test. * *p*<0.05.

Supplementary Figure 2 Quantification of cell types in AOM/DSS tumors. **(A)** Representative images of OLMF4^+^ immunohistochemistry (IHC) in AOM/DSS-induced tumors quantified by two different investigators blinded to genotype (one representative graph is shown). Yellow arrows highlight examples of OLMF4^+^ cells. Unpaired t-test. **p*<0.05. **(B)** Heatmap of *p* values of beta-distribution where a *p* value of <0.20 indicates a cell type is present. Each vertical bar represents one sample. HPF: high-power field.

Supplementary Figure 3 Characterization of intratumoral immune cells in orthotopic MC-38 tumor model. **(A)** Mice were orthotopically injected with 5x10^4^ MC-38 cells. Intratumoral FOXP3^+^ T cells were quantified by flow cytometry. **(B)** Intratumoral T cells were stimulated with PMA/ionomycin for 5 hours followed by intracellular cytokine staining with representative flow cytometry plots shown for CD8^+^ T cells (left) and quantification (right). **(C)** Quantification of intracellular cytokines for CD4^+^ T cells analyzed as in **(B)**. **(D)** Flow cytometry plots and quantification of intratumoral neutrophils and dendritic cells. Unpaired t-test or Mann-Whitney U-test.

Supplementary Figure 4 Characterization of intratumoral immune cells in a subcutaneous MC-38 tumor model. **(A)** Mice were injected SC with 5x10^4^ MC-38 cells with tumor volume quantified by a digital caliper. Some mice received paratumoral clodronate or control liposomes. ANOVA with *post-hoc* Šidáks. Data are pooled from 5 independent experiments. **(B)** Intratumoral T cells were stimulated with PMA/ionomycin for 5 hours followed by intracellular cytokine staining with CD8^+^ T cells quantified as well as **(C)** CD4^+^ T cells. **(D)** Frequency of intratumoral FOXP3^+^ Treg cells. **(E)** Quantified data from flow cytometry plots of intratumoral dendritic cells, **(F)** neutrophils, and **(G)** macrophages. Mann-Whitney U-test. **(B-G)** Data are pooled from two independent experiments.

Supplementary Figure 5 Stimulation of human tumoroids with recombinant IL-23 does not alter tumoroid growth **(A)** Treatment of human-derived healthy colorectal organoids and colorectal tumor organoids (“tumoroids”) with exogenous recombinant human IL-23. Time-lapse imaging and quantification of organoid and tumoroid size. Images are representative. Repeated measures ANOVA. **(B)** Representative pSTAT3 IHC in human colorectal cancer. Red arrows indicate pSTAT3-positive cells. Circles indicate epithelial cells in tumor section. Images taken at 10X magnification.
