# Supplementary material for: Distinct roles for interleukin-23 receptor signaling in regulatory T cells in sporadic and inflammation-associated carcinogenesis
Source: Front Oncol. 2024 Feb 5;13:1276743. doi: 10.3389/fonc.2023.1276743 (PMC10876294; doi:10.3389/fonc.2023.1276743)
Supplement: Supplementary file 1 [file Table_1.docx]

**Table S1 FC antibodies**

| Antigen-label | Source | Cat# |
| --- | --- | --- |
| CD25-BV421 | Biolegend | 102034 |
| CD25-APC | eBioscience | 17-0390-82 |
| CD45-BV785 | Biolegend | 103149 |
| CD3-PE-Cy7 | Biolegend | 100320 |
| CD44-PE-Cy5 | Biolegend | 103010 |
| CD4-FITC | Biolegend | 116003 |
| IL-4-PerCP/Cy5.5 | Biolegend | 504123 |
| IFNgamma-APC | Biolegend | 505810 |
| IL-17A-BV421 | Biolegend | 506926 |
| CD8-PerCP/Cy5.5 | Biolegend | 126609 |
| CD4-APC-Cy7 | Biolegend | 100414 |
| CD8-PE-Cy7 | Biolegend | 100722 |
| CD11b-FITC | Biolegend | 101206 |
| LY6G-APC | Biolegend | 127613 |
| CD103-PE | Biolegend | 121406 |
| MHCII-UV387 | Biolegend | 107669 |
| LY6C-PerCPCy5.5 | Biolegend | 128012 |
| CD11C-BV421 | Biolegend | 117330 |
| TNF-FITC | Biolegend | 506304 |
| MHCII-PECy5 | ThermoFisherScientific | 15532182 |
| CD45-SparkYG580 | Biolegend | 103171 |
| CD3-BV750 | Biolegend | 100373 |
| NK1.1-BV650 | Biolegend | 108735 |
| CD19-APCFire750 | Biolegend | 115557 |
| MHCII-PECy7 | Biolegend | 107630 |
| CD11c-BV421 | Biolegend | 117330 |
| CD11b-PerCP | Biolegend | 101229 |
| CD4-ApcFire810 | Biolegend | 100480 |
| CD45-AF594 | Biolegend | 103144 |
| CD8-SparkBlue550 | Biolegend | 100780 |
| FOXP3-PerCPCy5-5 | ThermoFisher Scientific | 45-5773-82 |
