# Supplementary figures and images for: Distinct roles for interleukin-23 receptor signaling in regulatory T cells in sporadic and inflammation-associated carcinogenesis
Source: Front Oncol. 2024 Feb 5;13:1276743. doi: 10.3389/fonc.2023.1276743 (PMC10876294; doi:10.3389/fonc.2023.1276743)

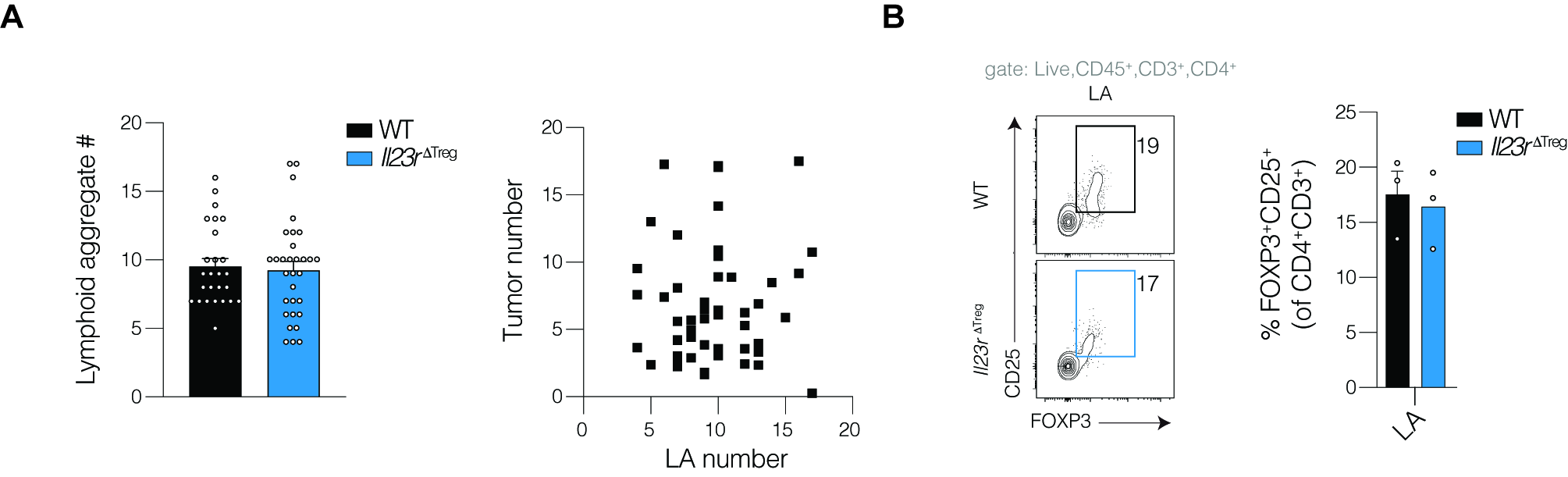

Supplement: Supplementary Figure 1 — Frequency of Treg cells in spleen and mLN is increased in Il23r ΔTreg mice following AOM/DSS. (A) Mice were injected with AOM/DSS as described in with quantification of colonic lymphoid aggregates (LA) shown. (B) Representative flow cytometry plots of Treg cells in LA (left) and frequencies (right). Unpaired t-test. * p<0.05. [file Image_1.tif]

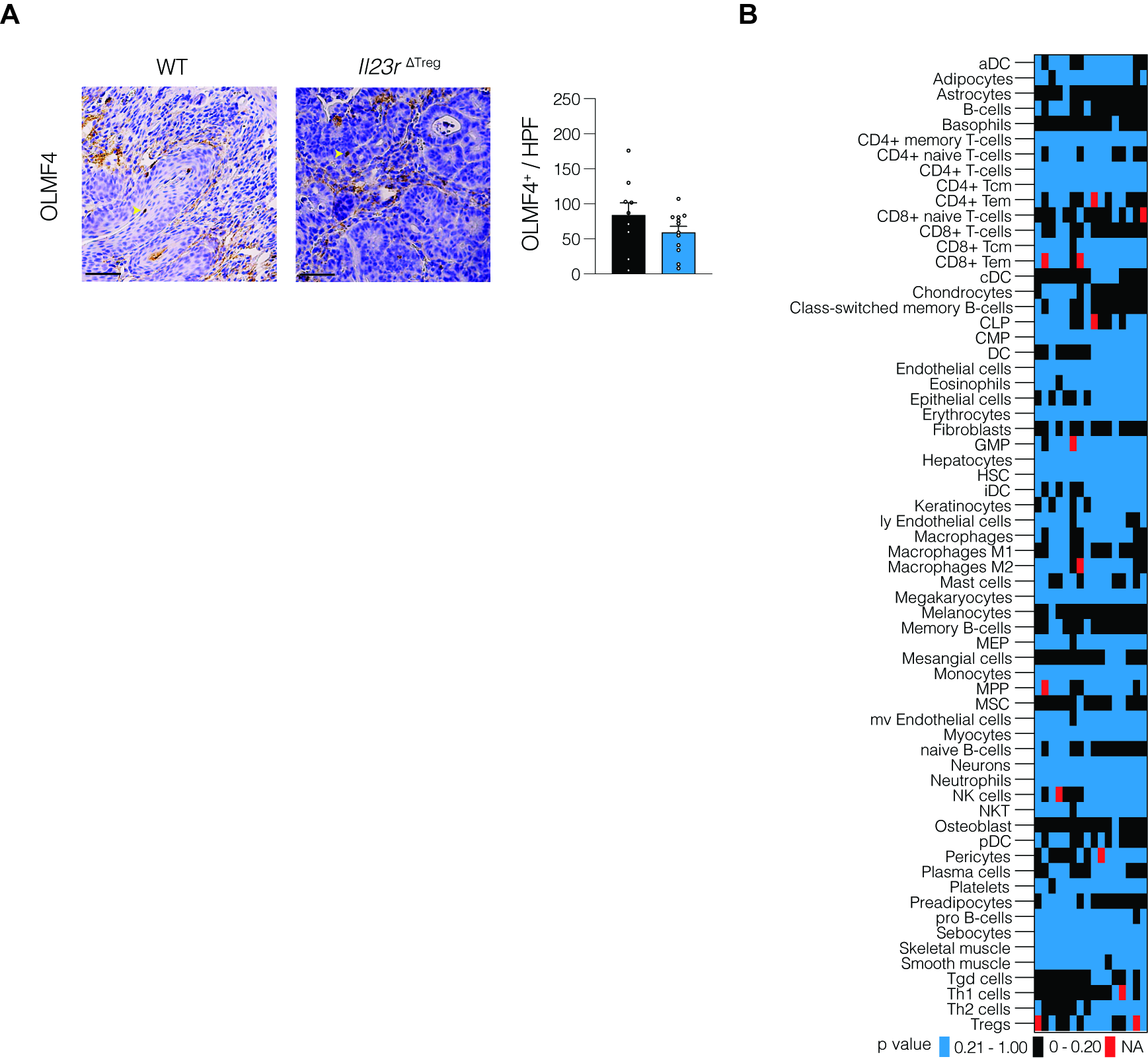

Supplement: Supplementary Figure 2 — Quantification of cell types in AOM/DSS tumors. (A) Representative images of OLMF4+ immunohistochemistry (IHC) in AOM/DSS-induced tumors quantified by two different investigators blinded to genotype (one representative graph is shown). Yellow arrows highlight examples of OLMF4+ cells. Unpaired t-test. *p<0.05. (B) Heatmap of p values of beta-distribution where a p value of <0.20 indicates a cell type is present. Each vertical bar represents one sample. HPF: high-power field. [file Image_2.tif]

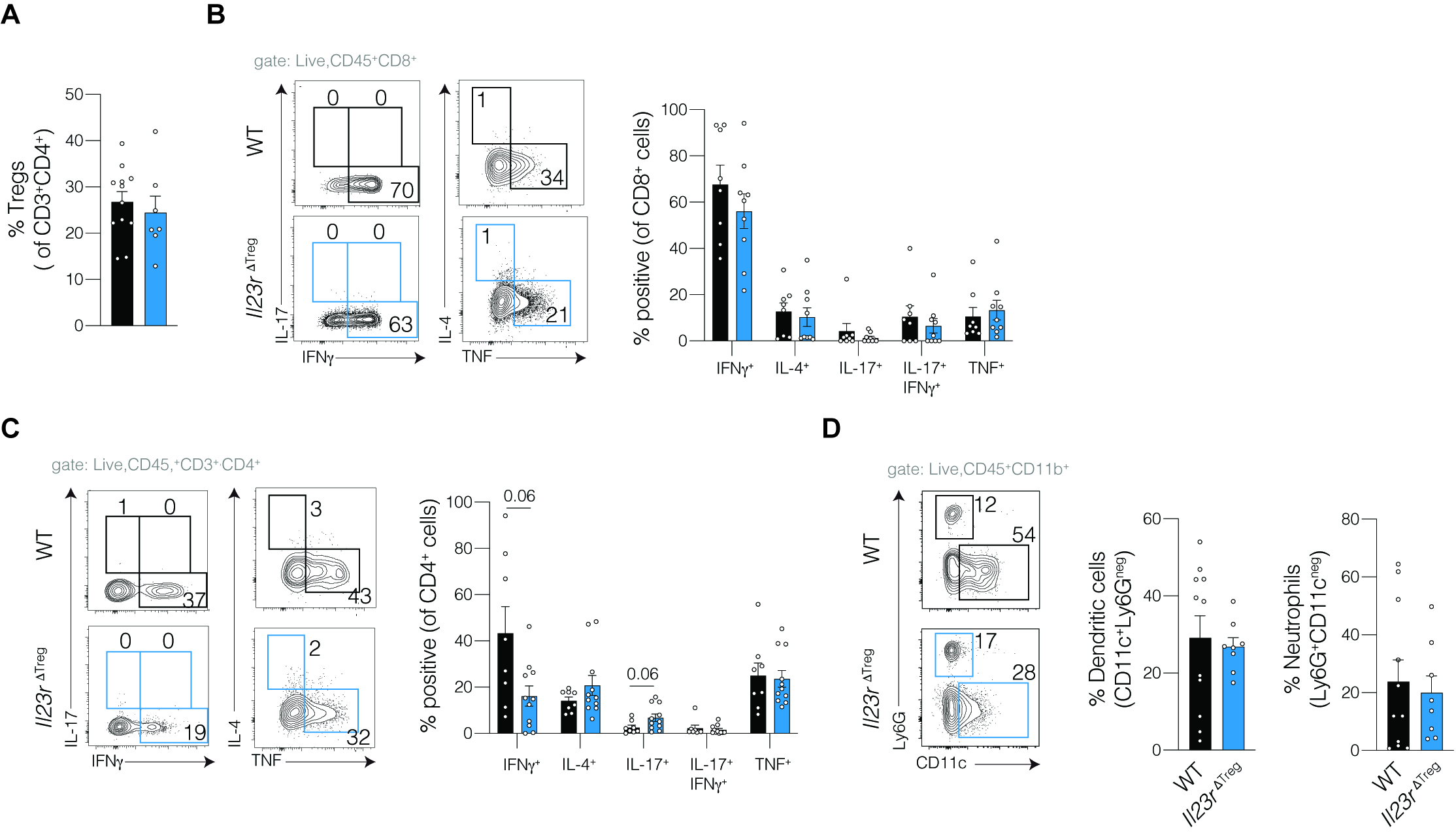

Supplement: Supplementary Figure 3 — Characterization of intratumoral immune cells in orthotopic MC-38 tumor model. (A) Mice were orthotopically injected with 5x10^4^ MC-38 cells. Intratumoral FOXP3+ T cells were quantified by flow cytometry. (B) Intratumoral T cells were stimulated with PMA/ionomycin for 5 hours followed by intracellular cytokine staining with representative flow cytometry plots shown for CD8+ T cells (left) and quantification (right). (C) Quantification of intracellular cytokines for CD4+ T cells analyzed as in (B). (D) Flow cytometry plots and quantification of intratumoral neutrophils and dendritic cells. Unpaired t-test or Mann-Whitney U-test. [file Image_3.tif]

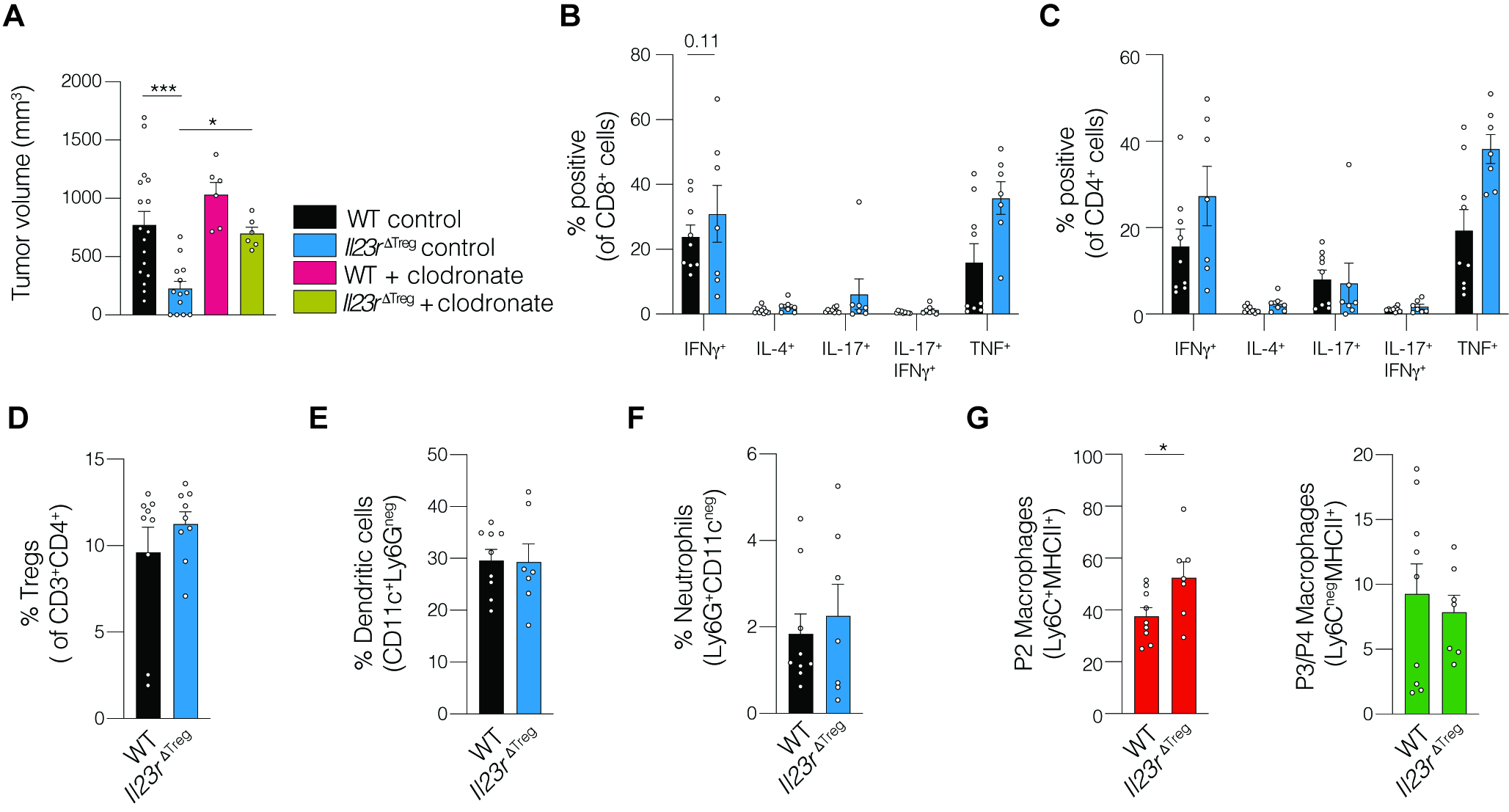

Supplement: Supplementary Figure 4 — Characterization of intratumoral immune cells in a subcutaneous MC-38 tumor model. (A) Mice were injected SC with 5x10^4^ MC-38 cells with tumor volume quantified by a digital caliper. Some mice received paratumoral clodronate or control liposomes. ANOVA with post-hoc Šidáks. Data are pooled from 5 independent experiments. (B) Intratumoral T cells were stimulated with PMA/ionomycin for 5 hours followed by intracellular cytokine staining with CD8+ T cells quantified as well as (C) CD4+ T cells. (D) Frequency of intratumoral FOXP3+ Treg cells. (E) Quantified data from flow cytometry plots of intratumoral dendritic cells, (F) neutrophils, and (G) macrophages. Mann-Whitney U-test. (B-G) Data are pooled from two independent experiments. [file Image_4.tif]

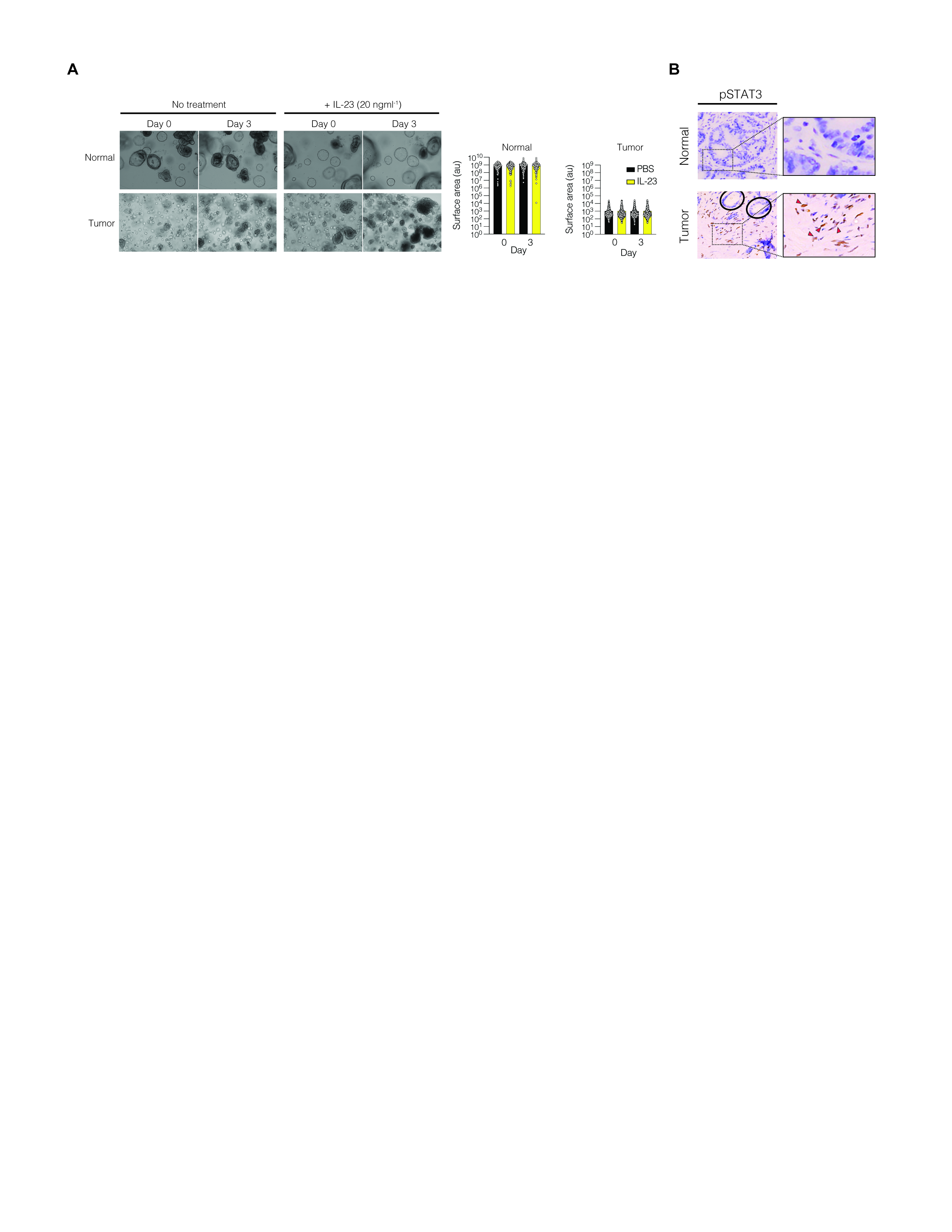

Supplement: Supplementary Figure 5 — Stimulation of human tumoroids with recombinant IL-23 does not alter tumoroid growth (A) Treatment of human-derived healthy colorectal organoids and colorectal tumor organoids (“tumoroids”) with exogenous recombinant human IL-23. Time-lapse imaging and quantification of organoid and tumoroid size. Images are representative. Repeated measures ANOVA. (B) Representative pSTAT3 IHC in human colorectal cancer. Red arrows indicate pSTAT3-positive cells. Circles indicate epithelial cells in tumor section. Images taken at 10X magnification. [file Image_5.tif]
